# Role of FLCN Phosphorylation in Insulin‐Mediated mTORC1 Activation and Tumorigenesis

**DOI:** 10.1002/advs.202206826

**Published:** 2023-04-21

**Authors:** Guoyan Wang, Lei Chen, Xinjian Lei, Senlin Qin, Huijun Geng, Yining Zheng, Chao Xia, Junhu Yao, Tong Meng, Lu Deng

**Affiliations:** ^1^ College of Animal Science and Technology Northwest A&F University Yangling Shaanxi 712100 China; ^2^ Department of Orthopedics Shanghai General Hospital School of Medicine Shanghai Jiaotong University Shanghai 200240 China

**Keywords:** FLCN phosphorylation, insulin, mTORC1, tumorigenesis

## Abstract

The amino acid‐stimulated Rag GTPase pathway is one of the main pathways that regulate mechanistic target of rapamycin complex 1 (mTORC1) activation and function, but little is known about the effects of growth factors on Rag GTPase‐mediated mTORC1 activation. Here, a highly conserved insulin‐responsive phosphorylation site on folliculin (FLCN), Ser62, that is phosphorylates by AKT1 is identified and characterized. mTORC2‐AKT1 is localized on lysosomes, and RagD‐specific recruitment of mTORC2‐AKT1 on lysosomes is identified as an essential step in insulin‐AKT1‐mediated FLCN phosphorylation. Additionally, FLCN phosphorylation inhibits the activity of RagC GTPase and is essential for insulin‐induced mTORC1 activation. Functionally, phosphorylated FLCN promotes cell viability and induces autophagy, and also regulates in vivo tumor growth in an mTORC1‐dependent manner. Its expression is also positively correlated with mTORC1 activity in colon cancer, clear cell renal cell carcinoma, and chordoma. These results indicate that FLCN is an important intermediate for cross‐talk between the amino acid and growth factor pathways. Further, FLCN phosphorylation may be a promising therapeutic target for diseases characterized by mTORC1 dysregulation.

## Graphical abstract

A mechanism by which FLCN phosphorylation in insulin‐mediated mTORC1 activation, which in turn achieves the crosstalk of amino acid‐mTORC1 and insulin‐mTORC1 axis. RagD‐specific recruitment of mTORC2‐AKT1 on lysosomes is an essential step in insulin‐AKT1‐mediated FLCN phosphorylation. FLCN phosphorylation inhibits the activity of RagC GTPase. Furthermore, clinical data in mTORC1‐associated tumors validate FLCN phosphorylation may be a promising therapeutic target.

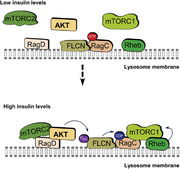

## 1 Introduction

Mechanistic target of rapamycin complex 1 (mTORC1) is the primary regulator of cell growth that plays an important role in the response to fluctuations in nutrient levels to maintain metabolic homeostasis.^[^ ^1^ ^]^ Once activated, mTORC1 integrates extracellular and intracellular signal inputs, such as amino acids, including *γ*‐aminobutyric acid (GABA),^[^ ^2^ ^]^ growth factors, stress, and energy states, through the phosphorylation of its downstream effectors, including ribosomal S6 kinase 1 (S6K), eukaryotic initiation factor 4E binding protein 1 (4EBP1), unc‐51‐like autophagy activating kinase 1 (ULK1), and transcription factor EB (TFEB), to regulate major cellular processes, such as growth, proliferation, and survival.^[^ ^3^ ^]^ mTORC1 dysregulation has been detected in many human diseases, including cancer and metabolic disorders.^[^ ^4^ ^]^

Both growth factors and amino acids significantly enhance mTORC1 activity through two different types of small GTPases—brain‐rich Ras homologs (Rheb) and Rag GTPase.^[^ ^5^ ^]^ Under growth factor signaling, Rheb, which is an allosteric activator of mTORC1,^[^ ^6^ ^]^ is activated and at least partially localized on the surface of the lysosome.^[^ ^6a^ ^]^ The binding of Rheb is required for the activation of mTORC1 by all signaling factors (including amino acids).^[^ ^6a^ ^]^ In contrast, Rag GTPase is thought to be an amino acid‐specific regulator of the mTORC1 pathway.^[^ ^7^ ^]^ The levels of specific amino acids (e.g., leucine and arginine) are sensed by different mechanisms and transduced through signal cascades.^[^ ^8^ ^]^ These processes eventually lead to the transformation of Rag GTPase into an active conformation, and the activated Rag heterodimer facilitates the translocation of mTORC1 to the surface of the lysosome, where the mTORC1 activator Rheb is located.^[^ ^9^ ^]^

There is a considerable amount of literature on the various mechanisms involved in the regulation of Rag activity and its role as a nutrient sensor.^[^ ^5a^ ^]^ One of the commonly reported mechanisms is the control of the nucleotide state of Rag mainly through guanine nucleotide exchange factors (GEFs) and GTPase‐activating proteins (GAPs). Some of the known GEFs and GAPs include the Ragulator,^[^ ^9b^ ^]^ the GATOR1 complex,^[^ ^10^ ^]^ and the leucyl‐tRNA synthetase (LeuRS, GAP, of RagD).^[8d^ ^]^ Of these, the most well‐known is the GAP follicle protein: follicle protein‐interacting protein complex (FLCN: FNIP2), which regulates the nucleotide state of the Rag protein, a tumor suppressor implicated in Birt–Hogg–Dubé hereditary cancer syndrome.^[^ ^7^, ^11^ ^]^ These known mechanisms that regulate FLCN‐Rag GTPase activity are all amino acid‐dependent, as mentioned earlier, and so far, little is known about the correlation of growth factors with FLCN‐Rag GTPase signaling.

In the current study, we explored the role of insulin, as a growth factor, in FLCN‐Rag GTPase signaling and mTORC1 activation. Our data highlight the important role of FLCN‐Rag GTPase in insulin‐regulated mTORC1 activation. We observed that in the absence of amino acids, insulin promotes the lysosomal localization of mTORC1 and activates the phosphorylation of downstream proteins of mTORC1 via regulation of the FLCN‐Rag GTPase pathway. These findings are the first to demonstrate the role of FLCN phosphorylation in insulin‐mediated mTORC1 activation.

## 2 Results

### 2.1 Insulin‐Regulated Lysosomal Localization of mTORC1

**Figure 1:**
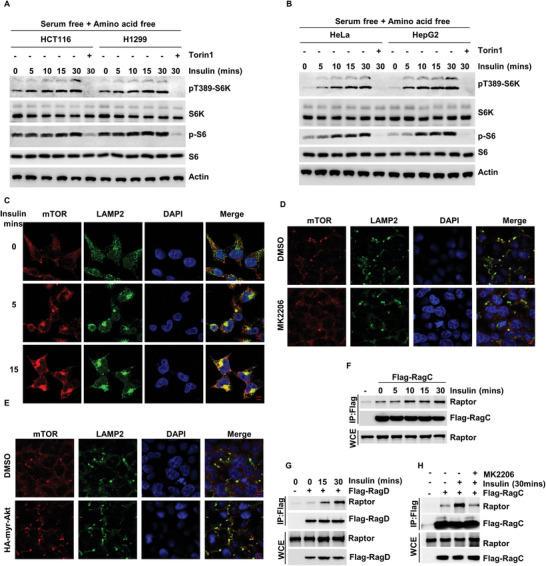
Insulin‐regulated lysosomal localization of mTORC1. A) HCT116, H1299, B) HeLa, HepG2 were starved of amino acids and serum for 24 h and then supplemented with insulin for 0, 5, 10, 15, or 30 min alone or in the presence of Torin1. The level of p‐S6K1, p‐S6 and indicated protein was analyzed via WB. C) HCT116 cells were starved of amino acids and serum for 24 h and then supplemented with insulin for 0, 5, or 15 min. Then, the cells were co‐immunostained for mTOR (red) and LAMP2 (green) and were visualized via confocal microscopy. D) HCT116 cells were treated with MK2206, or E) overexpressed HA‐myr‐Akt. Then, the cells were co‐immunostained for mTOR (red) and LAMP2 (green) and were visualized via confocal microscopy. HEK293T cells were starved of amino acids and serum for 24 h and then supplemented with insulin for 0, 5, 10, 15, or 30 min. The interaction of endogenous regulatory‐associated of mTOR (Raptor) and F) Flag‐RagC or G) Flag‐RagD were analyzed via a co‐IP assay. H) HEK293T cells were starved of amino acids and serum for 24 h and then supplemented with insulin for 30 min alone or in the presence of MK2206. The interaction of endogenous Raptor and Flag‐RagC were analyzed via a co‐IP assay.

To verify that insulin is able to activate the mTORC1 signaling pathway in the absence of amino acids, we pretreated cells from various cell lines with serum‐free and amino acid‐free medium and stimulated them with insulin. In HCT116, H1299, HeLa, and HepG2 cells, both insulin and epidermal growth factor (EGF) could activate the mTORC1 signaling pathway (**Figure** **1**A,B and Figure S1A,B, Supporting Information).

Since the main role of amino acids in the mTORC1 pathway is to mediate the lysosomal localization of mTORC1,^[^ ^1^ ^]^ we next examined the effects of insulin and EGF on the localization of mTORC1. We found that with insulin and EGF stimulation, the co‐localization of LAMP2 and mTOR increased significantly (Figure 1C and Figure S1C, Supporting Information). Moreover, on treatment of MK2206, an inhibitor of AKT, the co‐localization of lysosomal‐associated membrane protein 2 (LAMP2) and mTOR was significantly reduced (Figure 1D), while the overexpression of HA‐myr‐AKT, the continuously activated form of AKT, significantly promoted the co‐localization of LAMP2 and mTOR (Figure 1E).

Previous studies have shown that the lysosomal localization of mTORC1 is mainly regulated by Rag GTPase.^[^ ^5a^ ^]^ Therefore, we next examined whether insulin could regulate the activation of Rag GTPase, and the effect of insulin on the binding between Rag GTPase components. Our data showed that the binding of RagA with RagC, and RagB with RagD did not change (Figure S1D,E, Supporting Information), while the binding of RagC/RagD to Raptor was enhanced in response to insulin stimulation (Figure 1F,G), and the binding of RagC to Raptor was inhibited by MK2206 and Torin1 (an inhibitor of mTORC1 and mTORC2) (Figure 1H and Figure S1F, Supporting Information). These findings indicate that insulin promotes the lysosomal localization of mTORC1 by regulating the activation of Rag GTPase.

### 2.2 Insulin‐Dependent FLCN Phosphorylation at Ser62 by AKT

**Figure 2:**
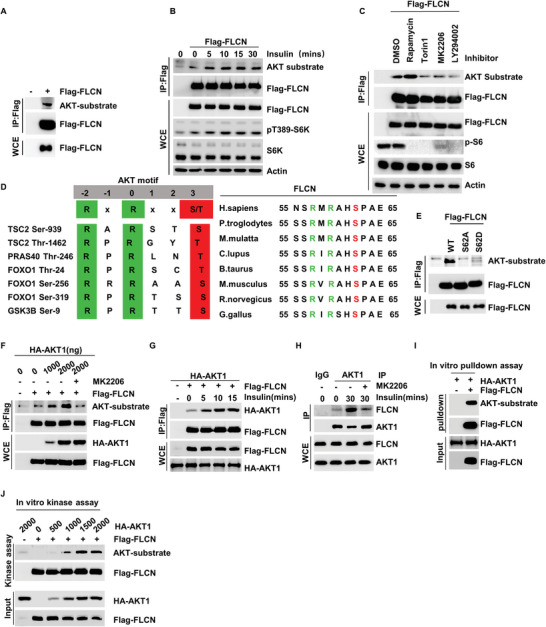
Insulin‐dependent FLCN phosphorylation at Ser62 by AKT Insulin‐regulated lysosomal localization of mTORC1. A) HEK293T cells were overexpressed Flag‐FLCN, immunoblotting with AKT‐substrate antibody to detect the phosphorylation of Flag‐FLCN. B) HEK293T cells were starved of amino acids and serum for 24 h and then supplemented with insulin for 0, 5, 10, 15, or 30 min, the phosphorylation of Flag‐FLCN was detected by AKT‐substrate antibody. C) Overexpression of Flag‐FLCN in HEK293T cells, and treatment of cells with different types of inhibitors, the phosphorylation of Flag‐FLCN was detected by AKT‐substrate antibody. D) A schematic showing the evolutionarily conserved putative AKT phosphorylation sites, Ser 62 within FLCN. E) Overexpression of the Flag‐FLCN, Flag‐FLCN S62A, or Flag‐FLCN S62D in HEK293T cells, the phosphorylation of Flag‐FLCN was detected by AKT‐substrate antibody. F) Overexpression of different concentrations of HA‐AKT1 or combined with MK2206 in HEK293T cells, the phosphorylation of Flag‐FLCN was detected by AKT‐substrate antibody. G) HEK293T cells were starved of amino acids and serum for 24 h and then supplemented with insulin for 0, 5, 10, or 15 min. The interaction of Flag‐FLCN and HA‐AKT1 were analyzed via a co‐IP assay. H) HEK293T cells were starved of amino acids and serum for 24 h and then supplemented with insulin for 30 min alone or in the presence of MK2206. The interaction of endogenous AKT1 and FLCN were analyzed via a co‐IP assay. I) Immunoprecipitates prepared from cells lysates were used in pulldown assays. In vitro pulldown assay indicated that HA‐AKT1 preferentially interacts with the Flag‐FLCN. J) Immunoprecipitates prepared from lysates cells were used in kinase assays. Immunoblotting with AKT‐substrate to detect the phosphorylation of Flag‐FLCN.

In order to elucidate the mechanism by which insulin promotes the lysosomal localization of mTORC1, we first screened the important proteins present upstream of Rag GTPase in the mTOR pathway. The results showed that both CASTOR1 and FLCN can be recognized by antibodies specific to the AKT substrate (**Figure** **2**A and Figure S2A–C, Supporting Information). Sequence analysis of these two proteins revealed that an AKT phosphorylation motif (RXRXXS/T) is present in CASTOR1 and is highly conserved among different species (Figure S2B, Supporting Information). Our data confirmed the regulatory effect of insulin on the phosphorylation of CASTOR1 at Ser14; in addition, the findings showed that the phosphorylation of Ser14 can regulate its binding to WDR24/59 in the GATOR2 complex, thereby regulating the activity of the mTORC1 pathway (Figure S2C–H, Supporting Information). Since data regarding the phosphorylation of CASTOR1 at Ser14 have already been published,^[^ ^12^ ^]^ the following work mainly focuses on the phosphorylation of FLCN.

We found that insulin stimulation can promote the phosphorylation of FLCN (Figure 2B), and this post‐translational modification of FLCN can be blocked by Torin1, LY294002 (an inhibitor of phosphatidylinositol 3‐kinase), and MK2206 (an inhibitor of AKT), but not by rapamycin (an inhibitor of mTORC1) (Figure 2C). These results further illustrate that the phosphorylation of FLCN is mediated by mTORC2‐AKT.

Through sequence analysis, we found that Ser62 of FLCN is a potential phosphorylation site of AKT (Figure 2D). To explore this possibility, we induced the mutation of Ser62(S) to Ala62(A) or Asp62(D) in FLCN, and our results showed that the AKT‐substrate antibody could not recognize the FLCN‐SA or FLCN‐SD mutant (Figure 2E).

In order to identify the kinase that induces FLCN phosphorylation, we screened previously reported kinases that can phosphorylate proteins with the RXRXXS/T motif, including AKT1, S6K1, and serum and glucocorticoid‐induced protein kinase 1 (SGK1),^[^ ^13^ ^]^ and our data showed that only AKT1 interacted with FLCN (Figure S2I,J, Supporting Information). Further, the phosphorylation of FLCN by AKT1 was found to be dose‐dependent (Figure 2F), and the binding of AKT1 to FLCN was found to be regulated by insulin and AKT1 activation (as MK2206 blocked the binding of AKT1 to FLCN) (Figure 2G,H and Figure S2K, Supporting Information). In addition, pulldown assay and in vitro kinase assay experiments showed that AKT1 can not only directly bind to FLCN, but also directly promote the phosphorylation of FLCN (Figure 2I,J). These results demonstrate that AKT1 can specifically bind to FLCN, thereby promoting phosphorylation of FLCN at Ser62 in an insulin‐dependent manner.

### 2.3 RagD‐Mediated Lysosomal Localization of mTORC2 and AKT1

**Figure 3:**
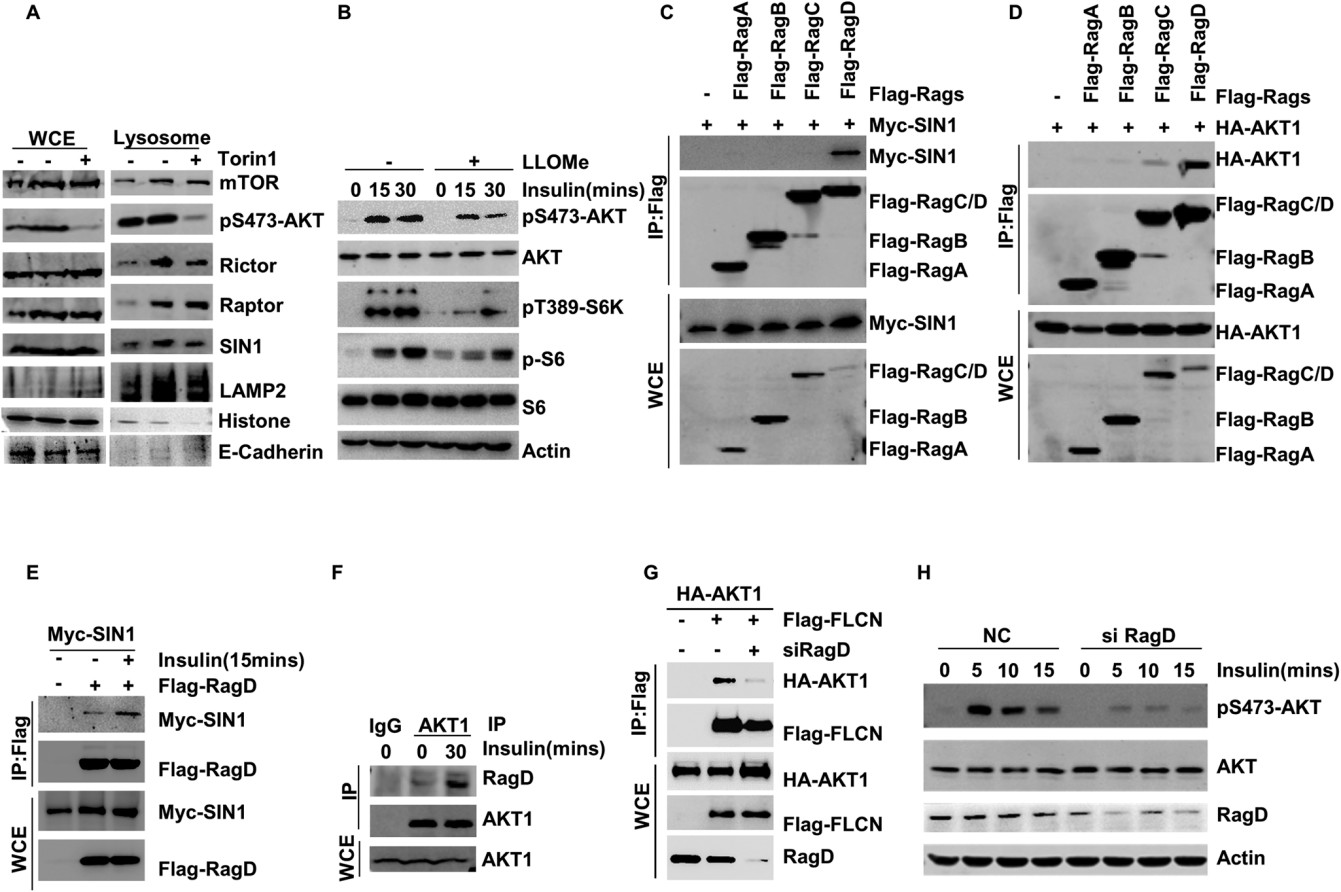
RagD‐mediated lysosomal localization of mTORC2 and AKT1. A) Lysosome was purified from HCT116 cells treated with or without Torin1. The indicated proteins were examined by WB. B) HCT116 cells were starved of amino acids and serum for 24 h and then supplemented with insulin for 0, 15, or 30 min alone or in the presence of LLOMe. The indicated proteins were examined by WB. C) The interaction of Flag‐RagA/B/C/D and Myc‐SIN1 were analyzed via a co‐IP assay. D) The interaction of Flag‐RagA/B/C/D and HA‐AKT were analyzed via a co‐IP assay. E) HEK293T cells were starved of amino acids and serum for 24 h and then supplemented with insulin for 15 min. The interaction of Flag‐RagD and Myc‐SIN1 were analyzed via a co‐IP assay. F) HCT116 cells were starved of amino acids and serum for 24 h and then supplemented with insulin for 30 min alone. The interaction of endogenous RagD and AKT were analyzed via a co‐IP assay. G) Knockdown the RagD in HCT116 cells, and the interaction of Flag‐FLCN and HA‐AKT were analyzed via a co‐IP assay. H) Knockdown the RagD in HCT116 cells, and phosphorylation of AKT and indicated protein were analyzed via WB.

Previous studies have shown that FLCN is a protein that is localized on lysosomes.^[^ ^14^ ^]^ To determine whether AKT1 is also localized on lysosomes, we isolated and purified intracellular lysosomes and found that not only Raptor, a component of mTORC1, but also Rictor and stress‐activated protein kinase interacting protein 1 (SIN1), components of the mTORC2 complex, were detected in the lysosomal samples (**Figure** **3**A). More importantly, we also detected the substrate of mTORC2, AKT1, and its activated form pS473‐AKT1, and the level of pS473‐AKT1 on lysosomes was significantly decreased in the presence of the mTORC2 inhibitor Torin1 (Figure 3A). In addition, we used LLOMe to induce lysosomal damage and found that LLOMe was able to inhibit mTORC1 and mTORC2 activities (Figure 3B). These findings imply that the mTORC2 pathway can be activated on lysosomes and that lysosomes are necessary for mTORC2 and AKT1 activity.

Rags (RagA/B/C/D) are critical small G proteases that mediate the localization of mTORC1 to the lysosome.^[^ ^14^ ^]^ We screened several Rags and found that SIN1, Rictor, and AKT1 could specifically bind to RagD (Figure 3C,D and Figure S3A,B, Supporting Information). AKT has several isoforms, namely, AKT1, AKT2, and AKT3. We found that RagD specifically binds to AKT1, but shows relatively weak binding to AKT2 and AKT3 (Figure S3C, Supporting Information). Co‐IP experiments revealed that SIN1, Rictor, and AKT1 can bind to RagD, and their binding is regulated by insulin. That is, insulin stimulation was found to enhance the binding of RagD to SIN1, rapamycin‐insensitive companion of mTOR (Rictor), and AKT1 (Figure 3E,F and Figure S3B, Supporting Information).

Next, we examined whether RagD was involved in the regulation of AKT1‐mediated phosphorylation of FLCN. We found that overexpression of RagD promoted the binding of AKT1 and FLCN (Figure S3D, Supporting Information), while knockdown of RagD blocked the binding of AKT1 and FLCN (Figure 3G). Moreover, we found that knockdown of RagD could inhibit the activation of mTORC2 (Figure 3H). These results suggest that RagD not only mediates the localization of mTORC2 and AKT1 on the lysosome, but also regulates AKT1‐mediated phosphorylation of FLCN.

### 2.4 Regulation of RagC Activity and Lysosomal Localization of mTORC1 by Phosphorylated FLCN

**Figure 4:**
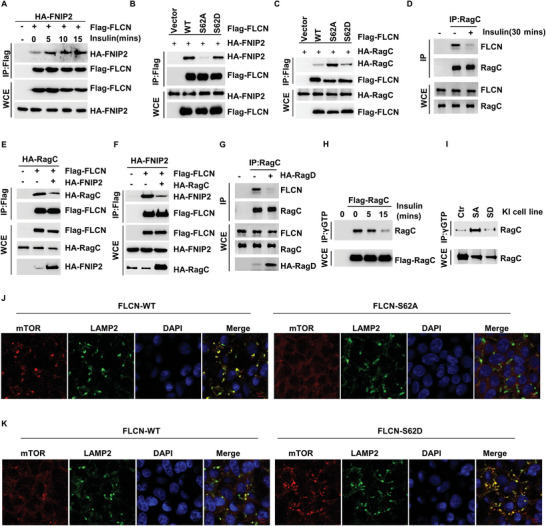
Regulation of RagC activity and lysosomal localization of mTORC1 by phosphorylated FLCN. A) HEK293T cells were starved of amino acids and serum for 24 h and then supplemented with insulin for 0, 5, 10, or 15 min. The interaction of HA‐FNIP2 and Flag‐FLCN were analyzed via a co‐IP assay. B) The interaction of Flag‐FLCN, Flag‐FLCN S62A, Flag‐FLCN S62D, and HA‐FNIP2 were analyzed via a co‐IP assay. C) The interaction of Flag‐FLCN, Flag‐FLCN S62A, Flag‐FLCN S62D, and HA‐RagC were analyzed via a co‐IP assay. D) HCT116 cells were starved of amino acids and serum for 24 h and then supplemented with insulin for 30 min. The interaction of endogenous RagC and FLCN were analyzed via a co‐IP assay. E) Overexpression of HA‐FNIP2 in HEK293T cells. The interaction of HA‐RagC and Flag‐FLCN were analyzed via a co‐IP assay. F) Overexpression of HA‐RagC in HEK293T cells. The interaction of HA‐FNIP2 and Flag‐FLCN were analyzed via a co‐IP assay. G) Overexpression of HA‐RagD in HEK293T cells. The interaction of HA‐RagC and Flag‐FLCN were analyzed via a co‐IP assay. H) HCT116 cells were starved of amino acids and serum for 24 h and then supplemented with insulin for 0, 5, 15 min, the GTP‐bound RagC was immunoprecipitated using GTP. I) The GTP‐bound RagC was immunoprecipitated using GTP in FLCN wild type (WT), FLCN S62A, FLCN S62D HCT116 cell line. J) FLCN WT, FLCN S62A, K) FLCN S62D HCT116 cell line were co‐immunostained for mTOR (red) and LAMP2 (green) and were visualized via confocal microscopy.

FLCN exhibits GAP activity, and this requires the formation of a complex with FNIP2.^[^ ^7^, ^11^ ^]^ We found that stimulation by insulin significantly enhanced the binding of FLCN to FNIP2 (**Figure** **4**A). To further demonstrate whether phosphorylation of FLCN regulates its binding to FNIP2, we treated cells with Torin1 and MK2206 and found that both inhibitors blocked the binding of FLCN to FNIP2 (Figure S4A, Supporting Information). More importantly, we found that FLCN‐SA lost its binding to FNIP2 (Figure 4B). These results suggest that phosphorylation of the FLCN Ser62 site is important for the binding of FLCN to FNIP2.

Next, we examined the role of phosphorylation of FLCN in its binding to RagC GTPase. We found that Torin1 promoted the binding of FLCN to RagC (Figure S4B, Supporting Information), whereas insulin stimulation inhibited the binding of FLCN to RagC (Figure S4C, Supporting Information). Our data show that the binding of FLCN‐SA to RagC is significantly stronger than the binding of FLCN‐WT and FLCN‐SD to RagC (Figure 4C), and our endogenous Co‐IP experiments demonstrate that insulin stimulation significantly inhibits the binding of FLCN to RagC (Figure 4D).

Since phosphorylation of FLCN plays diametrically opposing roles in its binding to RagC and FNIP2, we hypothesized that FNIP2 and RagC GTPase bind to FLCN in a competitive manner. To prove this hypothesis, we induced overexpression of FNIP2 and found that FNIP2 was able to inhibit the binding of FLCN to RagC (Figure 4E). Consistent with this result, overexpression of RagC was found to significantly inhibit the binding of FLCN to FNIP2 (Figure 4F).

Figure 3 demonstrates that RagD regulates AKT1‐mediated phosphorylation of FLCN, so we also examined the effect of RagD on the binding of FLCN to RagC. Our results show that overexpression of RagD inhibits the binding of FLCN to RagC GTPase (Figure 4G), while knockdown of RagD promotes the binding of FLCN to RagC GTPase (Figure S4D, Supporting Information). This implies that RagC GTPase and FLCN also bind to FLCN in a competitive manner.

Next, we examined the effect of phosphorylation on the stability of FLCN and found that phosphorylation of the FLCN Ser62 site had little effect on the stability of FLCN (Figure S4E, Supporting Information). Previous studies have shown that the main function of FLCN is to facilitate the conversion of RagC‐GTP to RagC‐GDP. Therefore, we examined the effect of FLCN phosphorylation on RagC activity and mTORC1 localization on lysosomes. We found that the activity of RagC was significantly attenuated with insulin stimulation by GTP beads (Figure 4H). To further illustrate this issue, we constructed FLCN‐SA‐ and FLCN‐SD‐knock in (KI) cell lines with CRISPR‐Cas9, and consistent with the results of exogenous overexpression, the activity of FLCN‐SA was found to be significantly higher than that of WT and FLCN‐SD (Figure 4I).

RagC GTPase activity plays an important role in the localization of mTORC1 on the lysosome.^[^ ^11a^ ^]^ Therefore, we next examined the regulatory effect of FLCN phosphorylation on mTORC1 localization on lysosomes by using FLCN‐SA‐ and FLCN‐SD‐KI cell lines, and we found that the co‐localization of mTOR with LAMP2 was significantly attenuated in FLCN‐S62A cells (Figure 4J), while FLCN‐S62D significantly enhanced the co‐localization of mTOR with LAMP2 (Figure 4K). These results suggest that phosphorylation of FLCN can regulate its binding to FNIP2 and RagC GTPase, inhibit the activity of RagC GTPase, and thus, promote the localization of mTORC1 on the lysosome.

### 2.5 Role of FLCN Phosphorylation in Insulin‐Mediated mTORC1 Activation

**Figure 5:**
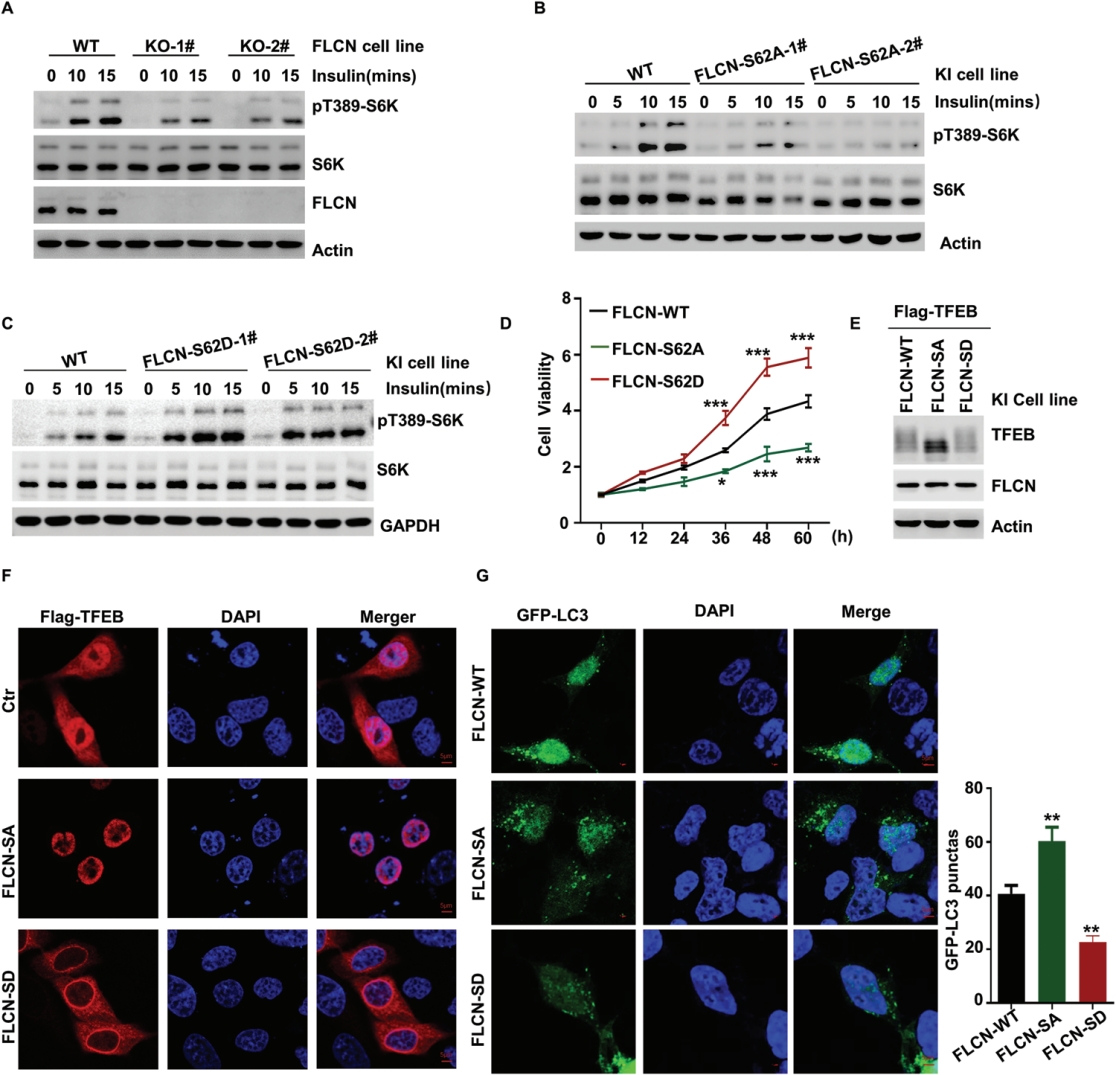
Role of FLCN phosphorylation in insulin‐mediated mTORC1 activation. A) FLCN KO‐1# and KO‐2# cell lines were starved of amino acids and serum for 24 h and then supplemented with insulin for 0, 5, 10, or 15 min. The indicated proteins were analyzed via WB. B) FLCN S62A‐1# and S62A‐2# cell lines were starved of amino acids and serum for 24 h and then supplemented with insulin for 0, 5, 10, or 15 min. The indicated proteins were analyzed via WB. C) FLCN S62D‐1# and S62D‐2# cell lines were starved of amino acids and serum for 24 h and then supplemented with insulin for 0, 5, 10, or 15 min. The indicated proteins were analyzed via WB. D) The cell viability of FLCN WT, FLCN S62A, and S62D cell line were detected via CCK8. Data were analyzed by two‐way ANOVA, and presented as the means ± SEM, *n* = 3; *p* value was considered statistically significant, ∗*p* < 0.05, ∗∗∗*p* < 0.001. E) The phosphorylation of TFEB and indicated protein were analyzed via WB in FLCN WT, FLCN S62A, and S62D cell line. F) Localization of TFEB in the nucleus and cytoplasm by immunofluorescence detection in FLCN WT, FLCN S62A, and S62D cell line. G) Detection of GFP‐LC3 puncta by immunofluorescence in FLCN WT, FLCN S62A, and S62D cell line. Statistical analysis of GFP‐LC3 puncta formation in each cell was performed on the indicated samples; Data were analyzed by one‐way ANOVA, and presented as the means ± SEM, *n* = 10; ∗∗*p* < 0.01.

To examine the function of FLCN in insulin‐mediated activation of mTORC1, we constructed FLCN‐knock out (KO) cell lines with CRISPR‐Cas9 and found that knockout of FLCN significantly inhibited insulin‐mediated activation of mTORC1 (**Figure** **5**A). To further demonstrate the function of FLCN phosphorylation in insulin‐mediated activation of mTORC1, we induced overexpression of FLCN‐WT, FLCN‐SA, and FLCN‐SD in FLCN‐knockdown cells and found that FLCN‐SA was able to inhibit insulin‐mediated activation of mTORC1, while FLCN‐SD was able to promote insulin‐mediated activation of mTORC1 (Figure S5A,B, Supporting Information). Consistent with these results, experiments with FLCN‐SA‐ and FLCN‐SD‐KI cell lines demonstrated that insulin‐mediated activation of mTORC1 was significantly inhibited in FLCN‐SA‐KI cell lines, whereas cell sensitivity to insulin was significantly enhanced in FLCN‐SD‐KI cell lines (Figure 5B,C). We also examined the effect of FLCN modification on cell viability, an important function downstream of mTORC1, and found that FLCN‐SA significantly inhibited cell viability, while FLCN‐SD significantly enhanced cell viability (Figure 5D and Figure S5C, Supporting Information).

Recent studies have shown that FLCN plays a central role in the phosphorylation of TFEB,^[^ ^11^, ^15^ ^]^ so we examined the role of phosphorylation of FLCN in the regulation of TFEB. Experiments with FLCN‐SA‐KI and FLCN‐SD‐KI cell lines showed that FLCN‐SA significantly inhibited the phosphorylation of TFEB (Figure 5E). In addition, FLCN‐SA promoted the localization of TFEB inside the nucleus, while FLCN‐SD caused the retention of TFEB in the cytoplasm (Figure 5F). These data demonstrate that phosphorylation of FLCN can regulate the localization of TFEB inside the cell, and TFEB can play an important role in cellular autophagy by regulating the expression of autophagy‐related genes. Therefore, we next examined the effect of phosphorylation of FLCN on cellular autophagy. Our analysis of GFP‐LC3 puncta revealed that FLCN‐SA was able to significantly promote autophagy, while FLCN‐SD was able to significantly inhibit autophagy (Figure 5G and Figure S5D, Supporting Information). These results suggest that phosphorylation of FLCN can regulate cellular sensitivity to insulin, promote the activation of mTORC1, inhibit the localization of TFEB inside the nucleus, suppress cellular autophagy, and promote cell viability.

### 2.6 In Vivo mTORC1‐Dependent Regulation of Tumor Growth by Phosphorylation of FLCN

**Figure 6:**
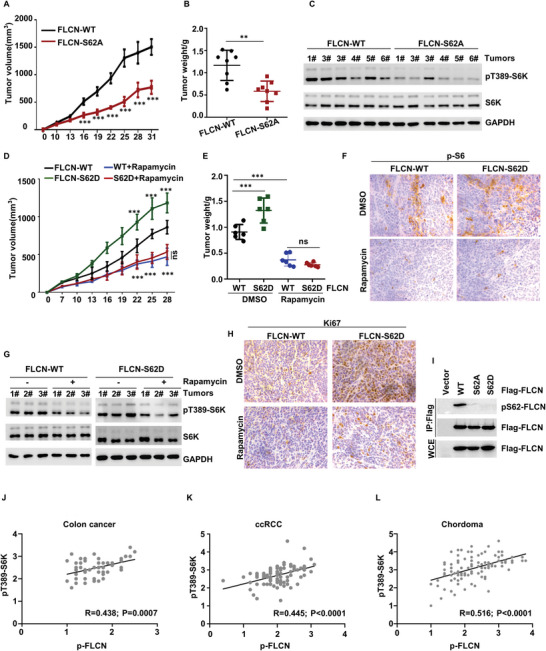
In vivo mTORC1‐dependent regulation of tumor growth by phosphorylation of FLCN. FLCN‐WT and FLCN‐S62A KI cells were injected into nude mice subcutaneous. The A) volume, B) weight of tumors, and C) mTORC1 activation in tumor samples were shown, Data were analyzed by one‐way ANOVA (B) or two‐way ANOVA (A), *p* value, and presented as the means ± SEM, *n* = 6; *p* value was considered statistically significant, ∗∗∗*p* < 0.001. FLCN‐WT and FLCN‐S62D KI cells were injected into nude mice subcutaneous alone or combined with rapamycin. The D) volume, E) weight of tumors, and F,G) mTORC1 activation and H) cell proliferation rate in tumor samples were shown, Data were analyzed by two‐way ANOVA (D, E), *p* value, and presented as the means ± SEM, *n* = 8; *p* value was considered statistically significant, ∗∗∗*p* < 0.001, ns denotes not significant. I) Overexpression of Flag‐FLCN, Flag‐FLCN S62A, Flag‐FLCN S62D in HEK293T cells. The proteins were enriched by IP assay and the specificity of anti‐phospho‐FLCN (S62) was detected by western blot. J–L) Correlation analysis of p‐FLCN and pT389‐S6K in colon cancer, J) *n* = 56, K) ccRCC, *n* = 88, L) chordoma, *n* = 113.

We next examined whether phosphorylation of FLCN is involved in in vivo mTORC1‐mediated tumorigenesis. Subcutaneous tumorigenic data showed that FLCN‐SA inhibited tumor growth and tumor volume (**Figure** **6**A,B), and the level of pT389‐S6K indicated that the inhibitory effect of FLCN‐SA on tumor growth was achieved mainly through inhibition of the mTORC1 pathway (Figure 6C). Moreover, FLCN‐SD‐KI cells showed a greater capacity for tumor formation and faster growth, both of which could be abolished by rapamycin treatment (Figure 6D,E). Western blot analysis of pT389‐S6K and immunohistochemistry of p‐S6 demonstrated that knock in of FLCN‐SD led to mTORC1 activation in tumors (Figure 6F,G), and immunohistochemistry of Ki67 showed that FLCN‐SD regulated tumor cell proliferation in an mTORC1‐dependent manner (Figure 6H). To further confirm the expression of FLCN Ser62 phosphorylation in mTORC1‐associated tumors, we generated a rabbit polyclonal antibody that could specifically recognize the FLCN phosphorylation (Figure 6I). mTORC1‐associated tumor samples, such as colon cancer, clear cell renal cell carcinoma (ccRCC) and chordoma, were also obtained and immunohistochemically stained for p‐FLCN and pT389‐S6K. Positive correlations were identified in all three tumor types (colon cancer: *R* = 0.438, *p* = 0.007, Figure 6J, Figure S6A, Supporting Information; ccRCC: *R* = 0.445, *p* < 0.0001, Figure 6K, Figure S6B, Supporting Information; chordoma: *R* = 0.516, *p* < 0.0001, Figure 6L, Figure S6C, Supporting Information), indicating the clinical relevance between FLCN phosphorylation and mTORC1 activity. Taken together, our results provide a possible therapeutic strategy of targeting that FLCN phosphorylation for mTORC1‐associated tumors.

In conclusion, these data suggest that phosphorylation of FLCN plays an important role in tumor growth through activation of mTORC1, thus illustrating the biological significance of FLCN phosphorylation in tumor growth.

## 3 Discussion

In this study, we identify a novel autoregulatory mTORC1 signaling pathway that is stimulated by insulin and mediated by phosphorylation of FLCN. Our current study highlights the crosstalk between insulin‐mTORC1 and amino acid‐mTORC1 axis, revealing that insulin achieves its interaction with the amino acid axis through phosphorylation of FLCN. Extensive studies have shown that the mTORC1 pathway is synergistically regulated by amino acids and growth factors,^[^ ^11b^ ^]^ and a growing body of evidence have uncovered the crosstalk between amino acids and growth factors. For example, the microspherule protein 1 (MCRS1), in an AA‐dependent manner, was reported to connect Rheb to mTORC1.^[^ ^16^ ^]^ In addition, tuberous sclerosis complex 2 (TSC2) was also found to be recruited to lysosomes by the Rag GTPases upon amino acid starvation, which was required for complete inactivation of mTORC1.^[^ ^17^ ^]^ Moreover, our recent work found that the cystine was involved in the regulation of the AKT‐mTORC1 axis by facilitating the phosphorylation of SIN1.^[^ ^18^ ^]^ These results highlight the critical roles of amino acid signals in the regulation of the growth factor‐mTORC1 axis. However, the existing studies on the amino acid‐mTORC1 axis dictated by growth factors are still limited. Although previous studies have shown that IGF can regulate mTORC1 lysosomal localization,^[^ ^19^ ^]^ the mechanisms involved are not clear. In present study, we found that insulin controlled the amino acid‐mTORC1 axis through promoting the phosphorylation of FLCN, which in turn achieved the crosstalk of amino acid‐mTORC1 and insulin‐mTORC1 axis. Furthermore, our clinical data in mTORC1‐associated tumors validated the correlation between FLCN phosphorylation and the classical mTORC1 substrate S6K. The novel model of mTORC1 pathway activation may be a promising therapeutic strategy for oncotherapy.

Mass spectrometry data for FLCN phosphorylation from other studies have demonstrated that FLCN can be phosphorylated at Ser62, but its activating kinase was not identified.^[^ ^20^ ^]^ Since Ser62 is embedded in the shared phosphorylation sequence of a member of the AGC family of kinases (RXRXXS/T, where X denotes any residue), it is possible that a member of the AGC family is the kinase that mediates FLCN S62 phosphorylation. Based on this speculation, we screened relevant kinases. Our results showed that FLCN did not bind to S6K1 and SGK1, and this is consistent with previous reports. Instead, FLCN was found to specifically bind to AKT1, and both in vitro and in vivo phosphorylation assays suggest that FLCN Ser62 is a direct target of AKT1.

Our findings showed that FLCN‐SA has a markedly increased binding capacity for RagC GTPase but a markedly weakened affinity for FNIP2, but we were not able to shed light on the reasons for this difference. One possible reason is that in growth factor‐deficient cells, FLCN is present on the lysosomal surface in an unphosphorylated and inactive form (FLCN S62A), where it is ready to activate RagC GTPase when growth factor levels are restored, as evidenced by the increased binding of FLCN S62A to RagC GTPase and diminished binding to FNIP2. However, this would require a mechanism to regulate FLCN‐FNIP2 GAP activity that is currently unclear. Alternatively, insulin may promote the phosphorylation of FLCN and its activity (FLCN S62D: FNIP2), activate RagC GTPase, and induce the release of RagC GTPase from the lysosome for recruiting mTORC1, as evidenced by the weaker binding of FLCN‐SD to RagC GTPase, but enhanced binding to FNIP2. In addition, there are probably other mechanisms to explain the effect of FLCN phosphorylation on mTORC1 activity. For example, it has been shown that the FLCN: FNIP2 complex is recruited to the lysosomal surface by binding to RagA/B‐GDP.^[^ ^11a^ ^]^ Therefore, the role of RagA/B in the regulation of mTORC1 activity by FLCN phosphorylation needs to be further investigated.

The role of lysosomes as important intracellular signaling hubs in the mTORC1 pathway is well known,^[^ ^5a^ ^]^ but little has been reported about their relevance to mTORC2 activation. However, it has been shown that mTORC2 activity is regulated by specific subcellular localization with strong spatial heterogeneity. For example, SIN1, Ras, and Rho are important regulatory proteins for mTORC2 plasma membrane localization and activation.^[^ ^21^ ^]^ Moreover, it has been shown that the distribution of intracellular lysosomes is closely related to the activity of the mTORC2 pathway,^[^ ^22^ ^]^ but the mechanisms underlying the localization of mTORC2 lysosomes are unclear. Fortunately, our data not only revealed that lysosomes are important organelles for mTORC2 activation, but also identified RagD as a key protein for mTORC2 lysosomal localization.

In addition to the clear role of RagC GTPase in promoting mTORC1 lysosomal recruitment,^[^ ^7a^ ^]^ this protein has a previously unrecognized function in the selective recruitment of specific mTORC1 substrates.^[^ ^7b^ ^]^ Recent studies have shown that FLCN‐mediated activation of RagC GTPase plays a key role in the phosphorylation of TFEB and other MiT‐TFE factors, but has no significant effect on the classical substrate S6K in the liver.^[^ ^7^, ^11^ ^]^ The discrepancy between our results and theirs can be explained by the inconsistency of upstream signaling. Both amino acid‐stimulated Rag activation and growth factor‐dependent Rheb activation are essential for mTORC1 activation and phosphorylation of its substrates. Thus, this “dual” mechanism of mTORC1 activation is known to be required for the phosphorylation of mTORC1 substrates such as S6K. However, the exacted upstream signal (amino acid or growth factor) was not clarified by Gosis et al.^[^ ^23^ ^]^ Although some studies specified the upstream signal, what their results elucidated was only the role of FLCN in amino acid‐mediated phosphorylation in TFE.^[^ ^11^, ^15^ ^]^ In contrast to these studies, our study focused on insulin, and the data showed that insulin promoted mTORC1 localization at the lysosome by phosphorylating FLCN in an amino acid‐independent manner. Consistent with the classical theory, our results showed that insulin significantly activates the phosphorylation of the classical substrate of mTORC1, S6K.

Moreover, the structure of RagA/C may also partially explain our results. The structure resolved from cryo‐electron microscopy shows the details of RagA/C binding to the mTORC1 subunit Raptor, highlighting the contribution of RagC in recruiting the lysosomal localization of mTORC1.^[^ ^24^ ^]^ S6K contains a TOR signaling (TOS) motif that is directly recognized and bound by mTORC1 via Raptor, allowing its recruitment and phosphorylation.^[^ ^7^, ^11^ ^]^ Consistently, our data showed that insulin positively regulated the phosphorylation S6K by promoting FLCN phosphorylation and inhibiting RagC activation. This is a novel, non‐classical mechanism for mTORC1 activation. In contrast, the TFE factor lacks a TOS pattern and instead contains a Rag binding site in its *N*‐terminal region.^[^ ^11b^ ^]^ The novel mechanism of mTORC1 substrate recruitment (TFE binding to Rag) may contribute to our finding that insulin‐mediated phosphorylation of FLCN and RagC activation also controlled TFE phosphorylation and nuclear localization. Thus, our results highlight that FLCN phosphorylation (or RagC activation) is not only essential for the recruitment of mTORC1 to the lysosome (S6K phosphorylation), but also acts as a novel mTORC1 substrate recruitment mechanism (TFE phosphorylation).

Consistent with these preliminary results, we found that FLCN‐SA not only significantly inhibited TFEB phosphorylation, but also promoted the localization of TFEB inside the nucleus. Moreover, FLCN‐SA was able to significantly promote autophagy, whereas FLCN‐SD had the opposite effect. These results suggest that FLCN phosphorylation‐mediated activation of RagC GTPase plays an important role in TFEB phosphorylation and cellular autophagy.

To conclude, the present findings demonstrate that insulin‐dependent phosphorylation of the highly conserved Ser62 residue on FLCN by AKT1 reduces its affinity with RagC, leading to an increase in RagC activity, which in turn promotes the lysosomal localization and activity of mTORC1. This mechanism ultimately affects mTORC1 activity‐dependent cell growth, autophagy, and tumor growth.

## 4 Experimental Section

### Antibodies and Reagents

The secondary antibodies were obtained from Sigma (Missouri, USA). The antibodies against pT389‐S6K (9234S/L), S6K (9202S), p‐S6 (4858S), S6 (2217S), pS473‐AKT (9271), AKT (9272), mTOR (2983S), Rictor (9476), Raptor (2280), SIN1 (D7G1A), FLCN (3697), TFEB (37785), WDR59 (53385), RagA (4357), RagC (9480), RagD (4470), AKT‐substrate (10001), Ki‐67 (12202S), LAMP2 (49067), Histone (4499), and E‐Cadherin (14472) were obtained from Cell Signaling Technology (MA, USA). Antibodies against Actin (20536‐1‐AP) and WDR24 (20778‐1‐AP) were obtained from proteintech. Antibodies against GAPDH (db106), anti‐Flag (db7002) and anti‐HA (db2603) were obtained from Hangzhou Bio Technology (Hangzhou, China). The pT389‐S6K (ab2571) for IHC was obtained from Abcam (Cambridge, UK). The anti‐phospho‐FLCN (S62) was generated by immunizing New Zealand rabbits with S62‐phosphorylated peptide corresponding to aa 59–68 (Arg‐Ala‐His‐Ser‐Pro‐Ala‐Glu‐Gly‐Ala‐Ser) of FLCN. The amino‐terminal of Keyhole Limpet Hemocyanin (KLH, H7017, Sigma) was conjugated with 4‐(*N*‐maleimide methyl) cyclohexane‐1‐succinimide carboxylate (SMCC, M5525, Sigma) to form a semi‐conjugated compound. The dissolved polypeptide was then slowly dripped into the semi‐conjugated compound and then the conjugate was injected subcutaneously into the rabbits by combining complete freundadjuvant (F5881, Sigma) and incomplete freundadjuvant (F5506, Sigma). Each rabbit was immunized with 500 µg–1 mg of immunogen. Antibodies were purified with the Thermo Fisher Antibody Purification kit. Finally, western blot was used to detect the specificity of the antibody. DMEM, RPMI 1640, fetal bovine serum (FBS), *β*‐mercaptoethanol, penicillin, and streptomycin were purchased from Gibco (Grand Island, USA). DMEM (amino acid‐free) was purchased from Genetimes Technology (Shanghai, China). Immobilized *γ*‐Amino‐hexyl‐GTP (AC‐117L) was obtained from Jena Bioscience (Jena, Germany). Cycloheximide (CHX, C7698), Torin1 (475991), Rapamycin (V900930), Insulin (I0310000), LLOMe (L7393), and EGF (SRP3027) were obtained from Sigma‐Aldrich (Missouri, USA). MK2206 (S1078) and LY294002 (S1105) were obtained from Selleck Chemicals (Shanghai, China). The Cell Counting Kit‐8 (CCK‐8) (K009) was purchased from ZETA LIFE (CA, USA).

### Cell Culture

HEK293T, HCT116, HeLa, and HepG2 cells were cultured in DMEM supplemented with 10% FBS at 37 °C in 5% CO_2_. H1299 cells were cultured in RPMI 1640 supplemented with 10% FBS at 37 °C in 5% CO_2_.

### Generation of the FLCN Knockout (KO) HCT116 Cell Lines

FLCN knockout cell lines were generated using lentiCRISPR methods.^[^ ^25^ ^]^ Briefly, guide RNA (sgRNA) was constructed into the lentiviral expression vector with Cas9 and sgRNA (lentiCRISPR). The lentiCRISPR vector was linearized using BsmBI. The sequence of sgRNA is:

sgRNA FLCN‐1#: CGGGCTGCTGGACTCGACGC.

sgRNA FLCN‐2#: CAGCCCGGGGCCCAAAAAGT.

### Generation of FLCN S62A/S62D Knockin (KI) HCT116 Cell Lines

FLCN S62A/S62D KI HCT116 cells were generated using CRISPR/Cas9. FLCN S62A/S62D specific sgRNA oligos were designed by the CRIPSR website (http://www.crispr.mit.edu/), the targeting sequence at S62 locus (5’‐CTCGACGCTGGCCCCCTCTG‐3’), the oligo donor sequences (S62A: 5’‐GTGGCATTCAGATGAACAGTCGGATGCGTGCGCACGCCCCCGCCGAGGGGGCCAGCGTCGAGTCCAGCAGCCCGGGGCCC‐3’; S62D: 5’‐GTGGCATTCAGATGAACAGTCGGATGCGTGCGCACGACCCCGCCGAGGGGGCCAGCGTCGAGTCCAGCAGCCCGGGGCCC‐3’). To generate FLCN S62A/S62D KI cell lines, the donor was inserted into pCDNA3.1 vector. 2 µg sgRNA and 2 µg 135‐bp homology arm‐containing donor plasmid were transfected into HCT116 cells. 24 h later, cells were treated with puromycin (2 µg mL^−1^). Then, the remained cells were separated into 96 well plate. After genomic DNA collection, PCR was performed (F’: 5’‐TGCACGGAGGTGCTGCAC‐3’; R’: 5’‐ACTGCTCTCAGGTCCTCC‐3’) and the products were sequencing.

### Plasmids

FLCN or CASTOR1 and its mutants were cloned into pCDNA3.1 vector such a Flag tag was fused to the N terminus. The rest of the plasmids were provided by P. Wang (Tongji University, Shanghai, China). All of the constructs were confirmed via DNA sequencing.

### siRNA Knockdown

Non‐specific control siRNA and siRNA for RagD were purchased from GenePharma (Shanghai, China). Cells were transfected with siRNA oligonucleotides using Lipofectamine. siRNA transfection of cells was performed according to the manufacturer's instructions. The following siRNA was used:

siRagD: 5’‐CTGTTCTTGGAGAGCACTAAT‐3’.

### Growth Factors Starvation and Re‐Stimulation

For insulin and EGF‐stimulation, cells were rinsed with PBS and incubated in amino acids and serum‐free DMEM medium for 24 h. Then, cells were stimulated with insulin and EGF for the indicated time.

### Immunoprecipitation (IP) and Western Blot

IP and western blot were performed as previously described.^[^ ^26^ ^]^ Transfected HEK293T cells were lysed in CHAPS lysis buffer (40 mm HEPES, pH 7.4, 120 mm NaCl, 1 mm EDTA, 10 mm *β*‐glycerophosphate, 0.3% CHAPS, and a cocktail of proteinase inhibitors).^[^ ^9b^ ^]^ After sonication for 10 min, the soluble fraction of the cell lysates was isolated via centrifugation at 12000 rpm in a microcentrifuge for 15 min at 4 °C. For IP, the cell lysates were centrifuged to remove the cell debris and then were incubated in HA‐conjugated beads (Abmart) or M2 beads (Sigma) for 2–3 h. Endogenous AKT or RagC was immunoprecipitated using an anti‐AKT or anti‐RagC polyclonal antibody. The beads were boiled after extensive washing; resolved via SDS‐PAGE gel electrophoreses, and analyzed via immunoblotting. The proteins were detected using the imaging System (Bio‐Rad, Hercules, CA, USA).

### Lysosomes Isolation

Lysosomes were isolated with lysosome Isolation Kit (Catalog Number LYSISO1, Sigma), all subsequent steps of the lysosomal isolation were performed according to manufacturer's description. In brief, the transfected HEK 293T cells were harvested on ≈90% confluency, and centrifuge the cells for 5 min at 600 *g*. add extraction buffer to break the cells in a 7 mL Dounce homogenizer using Pestle B (small clearance). After 20 strokes, the nuclei were removed by centrifugation at 1000 *g* for 10 min, the supernatant was centrifuged at 20 000 *g* for 20 min and the resulting pellet, containing the crude lysosomal fraction (CLF). To further enrich the lysosomes in the CLF, option C was used according to the manufacturer's protocol. The final pellet (lysosomal) fraction was prepared for immunoblotting.

### GTP‐Binding Assay

For binding of RagC to GTP‐Agarose beads, the HCT116 cells were harvested on ≈90% confluency. Suspended cells in binding buffer (20 mm HEPES pH 8, 150 nm NaCl, 10 mm MgCl_2_, and a cocktail of proteinase inhibitors) and lysed using three freeze thaw cycles, then centrifuged at 14 000 *g* and the supernatants were incubated with 100 µL of GTP‐Agarose suspension (G9768, Sigma Aldrich) for 1 h with rotation at 4 °C. The beads were pelleted by centrifugation, washed three times in binding buffer and suspended in 40 µL SDS‐PAGE sample buffer. The proteins were boiled, resolved via SDS‐PAGE gel electrophoreses, and analyzed via immunoblotting.

### Immunofluorescence Staining

The cells were washed three times with PBS and were fixed for 15 min at room temperature with 4% (vol/vol) paraformaldehyde and permeabilized with 0.2% Triton X‐100 for 20 min on ice. Following permeabilization, nonspecific binding in the cells was blocked by incubation for 30 min at room temperature with 1% BSA in PBS and cells were incubated overnight with specific primary antibodies. After PBS washed three times, cells were incubated for another 1 h with secondary antibodies (Alexa Fluor 488‐conjugated anti‐mouse IgG (A21202), Alexa Fluor 488‐conjugated anti‐rabbit IgG (A21206) or Alexa Fluor 555‐conjugated anti‐mouse IgG (A31570). Nuclei were counterstained with DAPI. All images were collected with a confocal microscope.

### Pulldown and In Vitro Kinase Assays

Immunoprecipitates prepared from lysates of HEK‐293T cells with the indicated antibodies (HA and Flag) were used in pulldown and kinase assays. The direct bound of FLCN and AKT1 was detected via Western blot. For kinase reaction immunoprecipitates were incubated in a final volume of 15 µL for 20 min at 37 °C in the kinase buffer (25 mm Hepes pH 7.5, 100 mm potassium acetate, 1 mm MgCl_2_) containing 500 µm ATP. The reaction was stopped by the addition of 200 µL ice‐cold Enzyme Dilution buffer (20 mm MOPS, pH 7.0, 1 mm EDTA, 0.01% Brij 35, 5% glycerol, 0.1% 2‐mercaptoethanol, 1 mg mL^−1^ BSA). Immunoblotting was used to detect the phosphorylation of FLCN at S62 in the kinase assays.

### Cell Viability Assay

FLCN WT, FLCN S62A, and FLCN S62D HCT116 cells were seeded in 96‐well plates at an initial cell density of 1500 cells per well in quintuplicate. Cell viability was assessed by CCK‐8 assay. Briefly, 100 mL of fresh medium containing 10% CCK8 reagent was given to replace the original medium for a 3‐h incubation at the temperature of 37 °C. Finally, the Synergy HT microplate reader (Bio‐Tek, USA) was used to determine the absorbance of each well at 450 nm. Each sample was performed in triplicate and each experiment was repeated at least three times independently.

### Autophagy Analysis

Autophagy analysis was conducted as the previous description.^[^ ^27^ ^]^ Briefly, GFP‐LC3 plasmid was used to transfect FLCN WT, FLCN S62A, and FLCN S62D HCT116 cells. Paraformaldehyde (4%) was used to fix the cells and maintained for 15 min. The permeabilized of fixed cells was performed using 0.5% Triton X‐100 in PBS for another 15 min. Sequentially, it was blocked at room temperature using 1% BSA for 1 h and stained with DAPI. Finally, the GFP‐LC3‐containing puncta were measured using laser a scanning confocal microscopy (Leica, Germany).

### Tumor Xenografts

Six‐week‐old male nude mice were obtained from Shanghai Experimental Animal Center (Shanghai, China). HCT116 cells were infected with retrovirus expressing vector, or lentivirus expressed FLCN WT, FLCN S62A, and FLCN S62D, and selected with 5 µg mL^−1^ puromycin in culture medium for 2 weeks. Then HCT116 cells were trypsinized into single cell suspensions and resuspended in PBS. ≈5 × 10^6^ HCT116 cells in 100 µL were injected into the right side and left dorsal flanks of each nude mouse, respectively. From 14 days after injection, the diameter of the tumor was measured every 2 days by a vernier caliper. Rapamycin was reconstituted in absolute ethanol at 10 mg mL^−1^ and diluted in 5% Tween 80 and 5% Peg‐400 before injection. Treatment was conducted by intraperitoneal injection of 1.5 mg kg^−1^ d^−1^ rapamycin for 5 consecutive days on day 6 after tumor cell injection, injections of carrier solution as controls. Tumor volume was calculated using the formula for volume: width^2^ × length × 0.5. Tumor size must not exceed 20 mm at the largest diameter in an adult mouse, according to the IACUC. The tumors were immunohistochemically stained using a kit from Dako (Copenhagen). Antibodies were presented in “4.1 Antibodies and reagents.” None of the experiments exceeded this limit in the study. All results were presented as mean ± SEM and *p < 0.05, **p < 0.01, ***p < 0.001; ns, not statistically significant by two tailed *t*‐test.

### Human Tissue Microarray

The tissue microarray (TMA) of chordoma samples were obtained from Shanghai General Hospital. Chordoma TMA comprised 113 samples from patients with histologically diagnosed chordoma. Colon cancer TMA comprised 56 samples from colon cancer patients. ccRCC TMA included 88 samples from ccRCC patients. The TMA sections were immunohistochemically stained using a kit from Dako (Copenhagen). Antibodies were presented in “4.1 Antibodies and reagents.” Immunostaining on each slide was assessed by two experienced pathologists with histochemistry score (H‐score). H‐score = Σ*pi* (*i* + 1) where *i* represents the intensity score and pi represents the percentage of cells with that intensity.

### Quantification and Statistical Analysis

GraphPad Prism 9.0 (GraphPad Software, Inc., La Jolla, CA, USA) software was used for data analysis. All experiments were repeated at least triple. Data were shown as mean ± SEM. Pairwise statistical significance was evaluated by two‐tailed Mann–Whitney *U*‐test or Student's *t*‐test. Statistical significance between multiple groups was evaluated by one‐way ANOVA or two‐way ANOVA with Fisher's LSD test or Bonferroni test for multiple comparisons. *p* value was considered statistically significant. In the graphed data *, **, and *** denote *p* values of < 0.05, 0.01, and 0.001, respectively. ns, not significant.

### Study Approval

This study was approved by the institutional review board of Shanghai General Hospital (approval number: 2021SQ013). The written informed consent for tissue and clinical data collection was signed by all patients or their legal guardians. All animal experiments were performed in compliance with the guide for the care and use of laboratory animals and were approved by the Institution Animal Care and Use Committee of the Northwest A&F University (NWAFU‐2020‐1131).

## Conflict of Interest

The authors declare no conflict of interest.

## Author Contributions

G.W.: Conceptualization, methodology, writing—original draft, investigation. L.C.: Conceptualization, methodology, writing—original draft, investigation, resources, supervision, project administration, and funding acquisition. X.L.: Conceptualization, writing—original draft, resources, supervision, project administration, and funding acquisition. S.Q.: Methodology, investigation. H.G.: Methodology, investigation. Y.Z.: Investigation. C.X.: Investigation. J.Y.: Resources, project administration, and funding acquisition. T.M.: Methodology, writing—original draft, investigation, resources, project administration, and funding acquisition. L.D.: Conceptualization, methodology, writing—original draft, investigation, writing—review and editing, resources, supervision, project administration, and funding acquisition. G.W., L.C., and X.L. are co‐first authors.

## Supporting information

Supporting Information

## Data Availability

The data that support the findings of this study are available in the supplementary material of this article.
