# Supplementary material for: Role of FLCN Phosphorylation in Insulin‐Mediated mTORC1 Activation and Tumorigenesis
Source: Adv Sci (Weinh). 2023 Apr 21;10(17):2206826. doi: 10.1002/advs.202206826 (PMC10265093; doi:10.1002/advs.202206826)
Supplement: Supplementary file 1 — Supporting Information [file ADVS-10-2206826-s001.pdf]

ADVANCED SCIENCE
Open Access

Supporting Information

for *Adv. Sci.*, DOI 10.1002/advs.202206826

Role of FLCN Phosphorylation in Insulin-Mediated mTORC1 Activation and Tumorigenesis

Guoyan Wang, Lei Chen, Xinjian Lei, Senlin Qin, Huijun Geng, Yining Zheng, Chao Xia, Junhu Yao, Tong Meng* and Lu Deng**

Figure S1 Insulin-regulated lysosomal localization of mTORC1

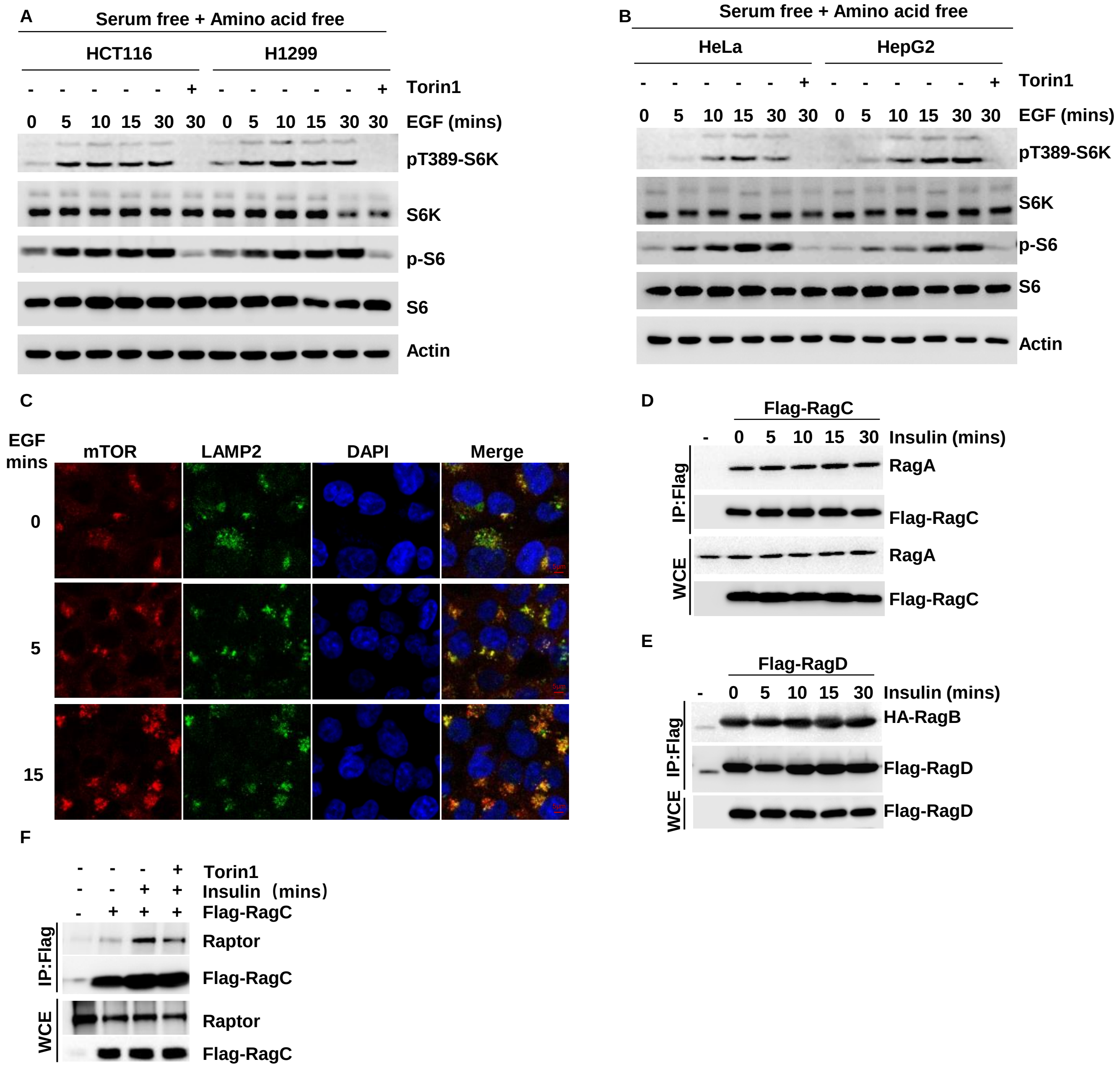

(A, B) HCT116, H1299 (A), HeLa, HepG2 (B) were starved of amino acids and serum for 24h and then supplemented with EGF for 0, 5, 10, 15, or 30 min alone or in the presence of Torin1. The level of p-S6K1, p-S6 and indicated protein was analyzed via WB.

(C) HCT116 cells were starved of amino acids and serum for 24h and then supplemented with EGF for 0, 5, 10, or 15 min. Then, the cells were co-immunostained for mTOR (red) and LAMP2 (green) and were visualized via confocal microscopy.

(D). HEK293T cells were starved of amino acids and serum for 24h and then supplemented with insulin for 0, 5, 10, 15, or 30 min. The interaction of endogenous RagA and Flag-RagC were analyzed via a co-IP assay.

(E). HEK293T cells were starved of amino acids and serum for 24h and then supplemented with insulin for 0, 5, 10, 15, or 30 min. The interaction of HA-RagB and Flag-RagD were analyzed via a co-IP assay.

(F). HEK293Twere starved of amino acids and serum for 24h and then supplemented with insulin for 30 min alone or in the presence of Torin1. The interaction of endogenous Raptor and Flag-RagC were analyzed via a co-IP assay.

Figure S2 Insulin-dependent FLCN phosphorylation at Ser62 by AKT

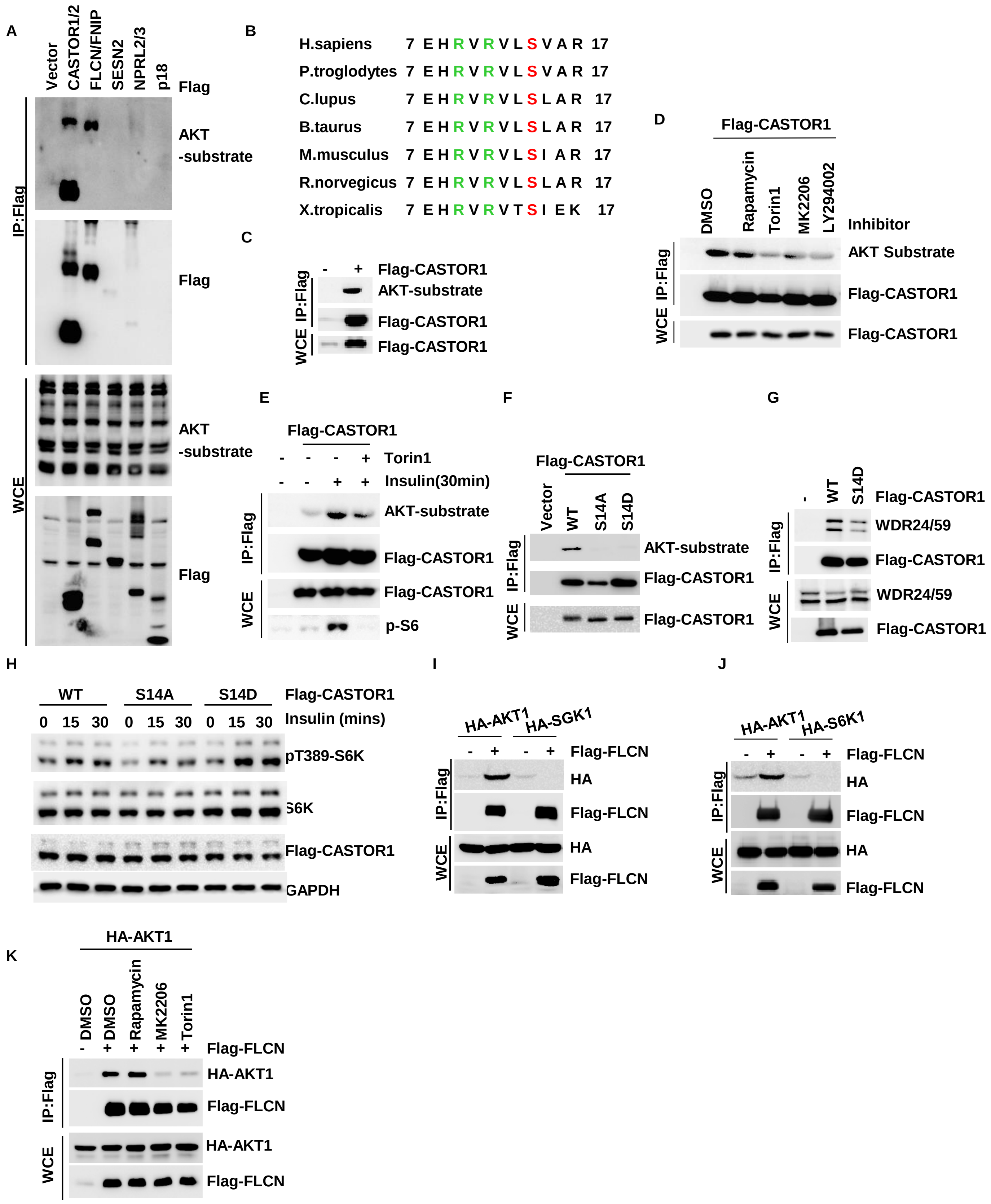

Figure S2 Insulin-dependent FLCN phosphorylation at Ser62 by AKT

(A). HEK293T cells were overexpressed different plasmids, immunoblotting with AKT-substrate and indicate antibody was used to detect the phosphorylation of different proteins.

(B). A schematic showing the evolutionarily conserved putative AKT phosphorylation sites, Ser 14 within CASTOR1.

(C). HEK293T cells were overexpressed Flag-CASTOR1, the phosphorylation of Flag-CASTOR1 was detected by AKT-substrate antibody.

(D). Overexpression of Flag-CASTOR1 in HEK293T cells, and treatment of cells with different types of inhibitors, the phosphorylation of Flag-CASTOR1 was detected by AKT-substrate antibody.

(E) HEK293T cells were starved of amino acids and serum for 24h and then supplemented with insulin for 30 min alone or in the presence of Torin1, the phosphorylation of Flag-CASTOR1 was detected by AKT-substrate antibody.

(F). Overexpression of the Flag-CASTOR1, Flag-CASTOR1 S14A or Flag-CASTOR1 S14D in HEK293T cells , the phosphorylation of Flag-CASTOR1 was detected by AKT-substrate antibody.

(G). The interaction of Flag-CASTOR1 and WDR24/59 were analyzed via a co-IP assay.

(H). Overexpression of Flag-CASTOR1, Flag-CASTOR1 S14A or Flag-CASTOR1 S14D in HEK293T cells were starved of amino acids and serum for 24h and then supplemented with insulin for 0, 15, or 30 min. The indicated protein were analyzed via WB.

(I). The interaction of endogenous HA-AKT1 or HA-SGK1 and Flag-FLCN were analyzed via a co-IP assay.

(J). The interaction of endogenous HA-AKT1 or HA-S6K1 and Flag-FLCN were analyzed via a co-IP assay.

(K). Overexpression of Flag-FLCN and HA-AKT in HEK293T cells, and treatment of cells with different types of inhibitors, the interaction of endogenous HA-AKT1 and Flag-FLCN were analyzed via a co-IP assay.

Figure S3 RagD-mediated lysosomal localization of mTORC2 and AKT1

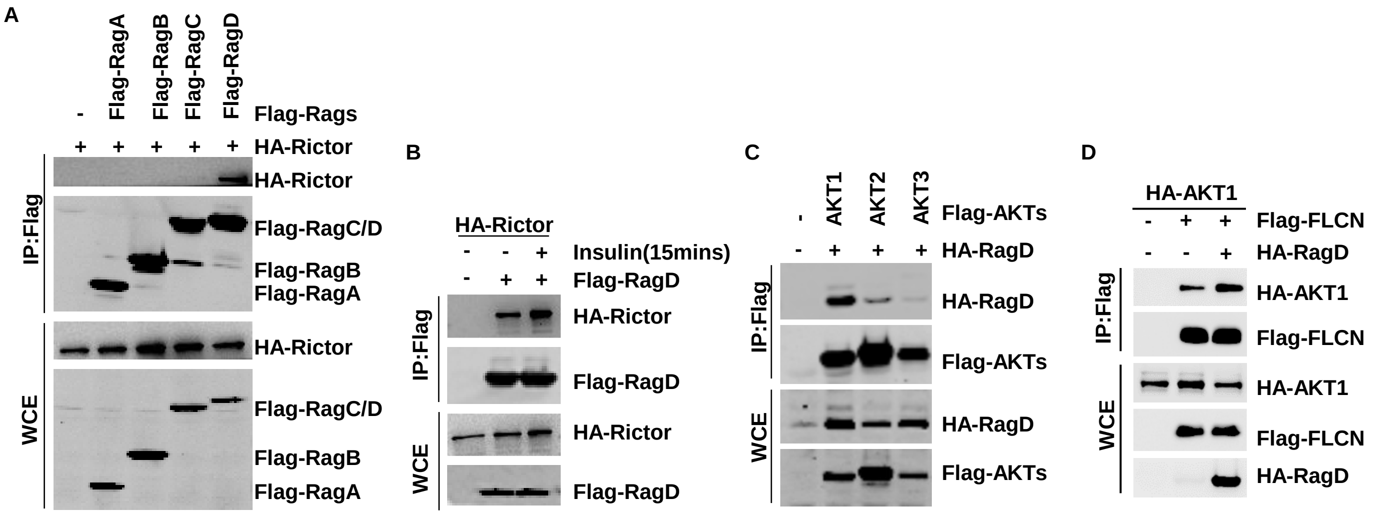

(A). The interaction of Flag-RagA/B/C/D and HA-Rictor were analyzed via a co-IP assay.

(B). HEK293T cells were starved of amino acids and serum for 24h and then supplemented with insulin for 15 min. The interaction of Flag-RagD and HA-Rictor were analyzed via a co-IP assay.

(C). The interaction of endogenous RagD and AKT1/2/3 were analyzed via a co-IP assay.

(D). Overexpression of HA-RagD in HEK293T cells, and the interaction of Flag-FLCN and HA-AKT were analyzed via a co-IP assay.

Figure S4 Regulation of RagC activity and lysosomal localization of mTORC1 by phosphorylated FLCN

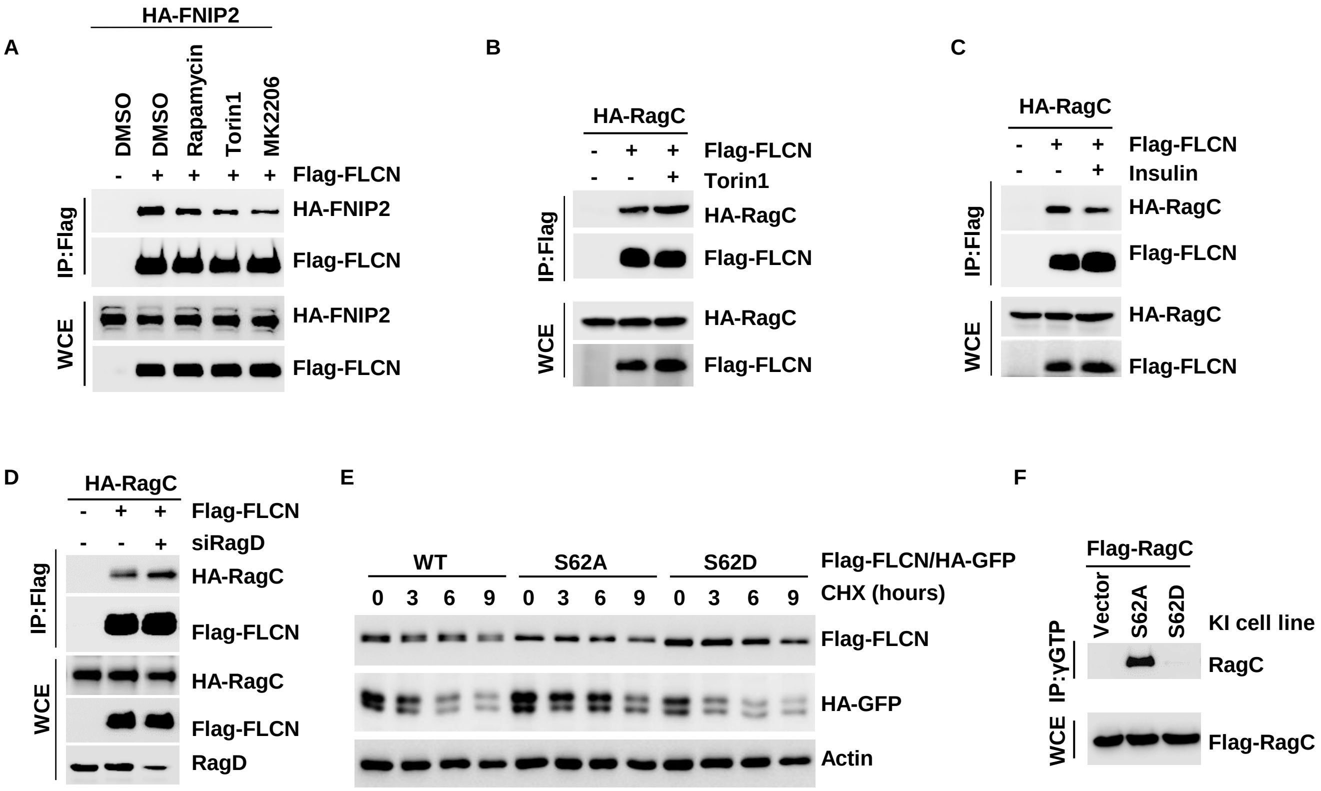

(A). Overexpression of Flag-FLCN and HA-FNIP2 in HEK293T cells, and treatment of cells with different types of inhibitors, the interaction of endogenous HA-FNIP2 and Flag-FLCN were analyzed via a co-IP assay.

(B). Overexpression of Flag-FLCN and HA-RagC in HEK293T cells, and treatment of cells with Totin1, the interaction of Flag-FLCN and HA-RagC were analyzed via a co-IP assay.

(C). HEK293T cells were starved of amino acids and serum for 24h and then supplemented with insulin for 30 min. The interaction of Flag-FLCN and HA-RagC were analyzed via a co-IP assay.

(D). Knockdown the RagD in HEK293T cells, and the interaction of Flag-FLCN and HA-RagC were analyzed via a co-IP assay.

(E). The stability of Flag-FLCN, Flag-FLCN S62A, and Flag-FLCN S62D were analyzed CHX assay.

Figure S5 Role of FLCN phosphorylation in insulin-mediated mTORC1 activation

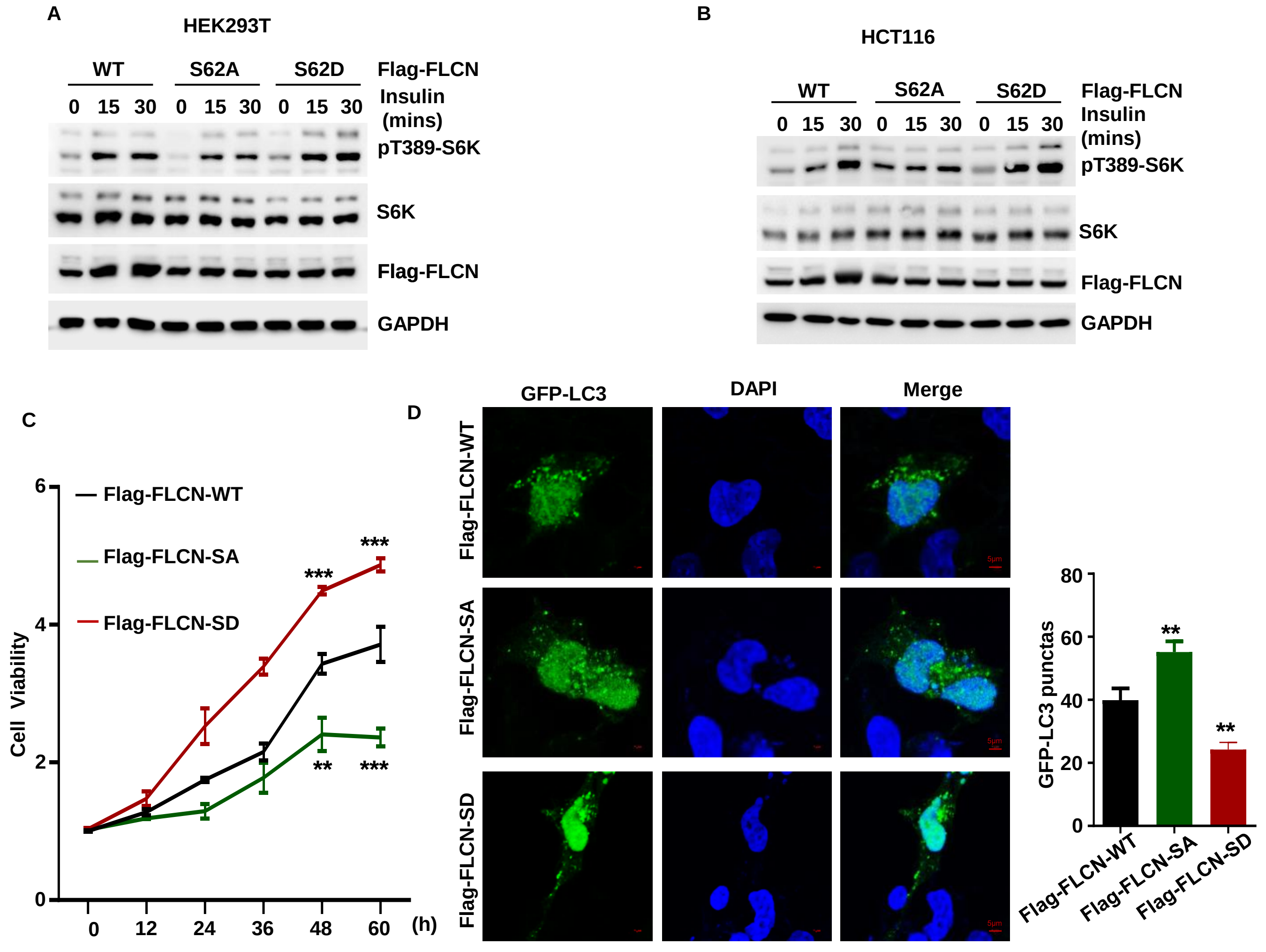

(A, B). Overexpression of Flag-FLCN, Flag-FLCN S62A, Flag-FLCN S62D in HEK293T (A) or HCT116 (B) cells, and cells were starved of amino acids and serum for 24h and then supplemented with insulin for 0, 15, or 30 min. The indicated protein were analyzed via WB.

(C). Overexpression of Flag-FLCN-WT, Flag-FLCN-S62A, Flag-FLCN-S62D in HCT116 cells, the cell viability were detected via CCK8, Data were analyzed by two-way ANOVA, and presented as the means ± SEM, n = 3; p value was considered statistically significant, $**p < 0.01$, $***p < 0.001$.

(D). Overexpression of Flag-FLCN-WT, Flag-FLCN-S62A, Flag-FLCN-S62D in HCT116 cells, the GFP-LC3 puncta were detected by immunofluorescence. Statistical analysis of GFP-LC3 puncta formation in each cell was performed on the indicated samples.

Figure S6 In vivo mTORC1-dependent regulation of tumor growth by phosphorylation of FLCN

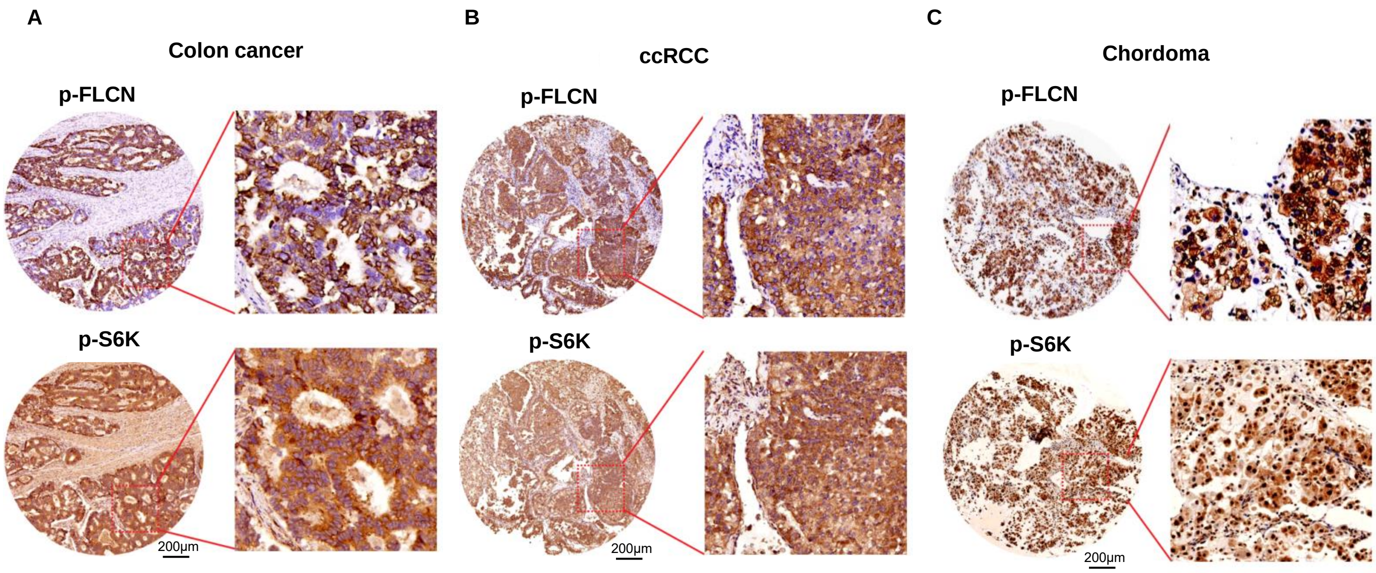

(A-C). Immunohistochemical staining for p-FLCN or p-S6K in colon cancer, n = 56 (A), Clear cell renal cell carcinoma (ccRCC), n = 88(B), chordoma, n = 113 (C), indicate p-FLCN or p-S6K levels in representative tumor tissues.
